# Descriptive Epidemiology and Whole Genome Sequencing Analysis for an Outbreak of Bovine Tuberculosis in Beef Cattle and White-Tailed Deer in Northwestern Minnesota

**DOI:** 10.1371/journal.pone.0145735

**Published:** 2016-01-19

**Authors:** Linda Glaser, Michelle Carstensen, Sheryl Shaw, Suelee Robbe-Austerman, Arno Wunschmann, Dan Grear, Tod Stuber, Bruce Thomsen

**Affiliations:** 1 Minnesota Board of Animal Health, St. Paul, Minnesota, United States of America; 2 Wildlife Health Program, Minnesota Department of Natural Resources, Forest Lake, Minnesota, United States of America; 3 Veterinary Services, Animal and Plant Health Inspection Service, United States Department of Agriculture, Madison, Wisconsin, United States of America; 4 National Veterinary Services Laboratories, Veterinary Services, Animal and Plant Health Inspection Service, United States Department of Agriculture, Ames, Iowa, United States of America; 5 Department of Veterinary Population Medicine, Veterinary Diagnostic Laboratory, College of Veterinary Medicine, University of Minnesota, St. Paul, Minnesota, United States of America; 6 Centers for Epidemiology and Animal Health, Veterinary Services, Animal and Plant Health Inspection Service, United States Department of Agriculture, Fort Collins, Colorado, United States of America; University of Minnesota, UNITED STATES

## Abstract

Bovine tuberculosis (bTB) was discovered in a Minnesota cow through routine slaughter surveillance in 2005 and the resulting epidemiological investigation led to the discovery of infection in both cattle and white-tailed deer in the state. From 2005 through 2009, a total of 12 beef cattle herds and 27 free-ranging white-tailed deer (*Odocoileus virginianus*) were found infected in a small geographic region of northwestern Minnesota. Genotyping of isolates determined both cattle and deer shared the same strain of bTB, and it was similar to types found in cattle in the southwestern United States and Mexico. Whole genomic sequencing confirmed the introduction of this infection into Minnesota was recent, with little genetic divergence. Aggressive surveillance and management efforts in both cattle and deer continued from 2010–2012; no additional infections were discovered. Over 10,000 deer were tested and 705 whole herd cattle tests performed in the investigation of this outbreak.

## Introduction

*Mycobacterium bovis* is the causative agent of bovine tuberculosis (bTB) and an important zoonotic disease. The United States initiated a State-Federal Bovine Tuberculosis (bTB) Eradication Program in 1917 and has one of the world’s lowest bTB prevalences in cattle. Minnesota participated in the national eradication program since its inception and gained TB Free status in 1976, five years after the last TB infected cattle herd in the state was eliminated. With over thirty years without any known bTB infection in cattle, the 2005 discovery of a bTB infected animal from Minnesota identified through slaughter surveillance was unexpected.

This paper documents the identification, testing, and epidemiology of an outbreak of bTB in beef cattle and white-tailed deer in Minnesota and the relationship between herds, and herds and deer based on cattle movement, infected deer locations, herd testing, and whole genome sequencing (WGS) of isolates from infected animals. There has been little documentation of molecular epidemiological investigations in bTB outbreaks that involve cattle and wildlife in the literature; this article provides a level of detail and data analyses not reported previously. The whole genome sequencing (WGS) technology supports the introduction and transmission theories of the epidemiological investigation in Minnesota. This information will serve to assist regulators, cattle producers, and epidemiologists in future bTB outbreaks.

## Materials and Methods

### Slaughter Surveillance

This introduction of bTB into Minnesota was identified through routine slaughter surveillance for bTB conducted by US Department of Agriculture (USDA) personnel at all US federally inspected slaughter plants. USDA veterinarians are trained to identify lesions in bovine carcasses that may be compatible with bTB infection and submit samples of lesions and associated lymph nodes from the animal for diagnostic testing to the National Veterinary Services Laboratories (NVSL) in Ames, Iowa. Carcasses with suspect lesions are held until diagnostic tests are completed. Positive tests result in condemnation of the carcass for food.

### Epidemiological Investigation

All positive slaughter samples are traced back to a herd of origin through verification of the animal’s identification, sales records, and DNA testing. Subsequent investigation methods of a positive slaughter animal are outlined in the state—federal cooperative bovine eradication program document, Bovine TB Uniform Methods and Rules (UMR) [1]. This includes requirements for tracing cattle movement in and out of infected herds, testing contact herds and trace herds, and area testing requirements when wildlife species are found infected with bTB. This live animal testing was done under Board of Animal Health statutory authority 35.05(d).

The epidemiological investigation in Minnesota included quarantine and bTB testing of all cattle or bison herds with fence line contact with an infected herd and all herds that may have bought or sold animals from the herd over a designated span of time. The number of years required for tracing cattle movement in any herd varied based on the age of animals found infected in that herd, but did not exceed seven years for any herd due to the lack of records available. Both private and sale barn sales were traced to the extent feasible. Animals sold for slaughter or feeding did not have animal identification recorded on sales documents so specific animals could not be traced. Often these animals were commingled into larger groups so the group was traced even as it was subdivided and resold to multiple owners. If trace animals were found in a herd in Minnesota, the animals were indemnified, euthanized and tested for bTB. If no trace animals remained in the herd, the herd was tested for bTB using live animal testing and the exposed animals were traced through the subsequent owner’s records of movement. Producers that sold animals to bTB infected herd owners had their herds quarantined and tested for bTB. When traces led to other states, the State Veterinarian and the USDA Area Veterinarian in Charge for each state were notified.

When bTB infection is found in wildlife, additional surveillance and testing requirements for livestock are required as outlined in the Bovine TB UMR [1]. These requirements include testing livestock in a geographic area as movement of deer between herds could spread bTB to nearby cattle. In early 2006, ten-mile radius circles were established around each of the five TB infected cattle herds and the livestock herds found within these areas were quarantined and tested for TB. Typically, cattle herds were tested in the fall each year after animals returned from pasture. Animals were likely to be handled at that time for other reasons including separating calves from their dams, dehorning or castrating, and vaccination or deworming.

Another component of the epidemiologic investigation was to determine if wildlife species were involved in the outbreak. White-tailed deer are susceptible to bovine bTB infection and were present at 2–3 deer/km^2^ in the area where the positive beef cattle herds were identified. They were known to feed in areas also used by cattle for feeding [2]. Given these factors, wildlife surveillance was targeted toward white-tailed deer. Initially in 2005, the Minnesota Department of Natural Resources collected samples from hunter-harvested deer. As the outbreak continued, additional methods were utilized to obtain samples from deer, including liberalization of hunting seasons, landowner shooting permits, and agency-sponsored culling.

### Live Animal Testing

All cattle twelve months of age and older were tested using the caudal fold tuberculin (CFT) test as described for a ‘whole herd test’ in the Definitions section of the Bovine TB UMR [1]. The intradermal injection of USDA Veterinary Services (VS) approved purified protein derivative (PPD) tuberculin bovis in the right or left caudal fold was palpated and observed within 72 ±6 hours of the injection time. Any increase in caudal fold thickness either observed or palpated at the site of injection is considered a response. This testing was performed by Bovine TB Certified veterinarians (USDA Accredited veterinarians that had received additional training in bovine tuberculosis from the Board of Animal Health) or by regulatory veterinarians. This live animal testing was done under Board of Animal Health statutory authority; MINN. STAT. 35.05 (2014). The comparative cervical tuberculin (CCT) test [1] was the follow up test used for any CFT test responders (suspects) in cattle. The CCT test was always performed by a regulatory veterinarian using balanced PPD tuberculin bovis and avian approved by USDA VS. Skin thickness is measured with a USDA VS approved dermal thickness gauge (caliper) before the injection and 72 ±6 hours after the injection and the change in thickness is plotted on the scattergram VS Form 6-22D to interpret the test result and classify the animal as Negative, Suspect or Reactor. Animals classified as Suspects can be retested after 60 days as an option for confirming a diagnosis. During this outbreak, owners were offered indemnity for Suspects so the animal could be euthanized and inspected or necropsied with diagnostic testing to confirm a diagnosis. Animals classified as Reactors must be indemnified and euthanized and inspected or necropsied with diagnostic testing to confirm a diagnosis [1}.

### Diagnostic Testing

#### Cattle

Necropsies were performed by state and federal veterinarians on cattle for evidence of bTB. Necropsy procedures included palpation and incision of retropharyngeal, mandibular and parotid lymph nodes from the head; tracheobronchial and mediastinal lymph nodes from the thorax; palpation of lungs and parietal pleura; incision of gastrohepatic, cranial and caudal mesenteric lymph nodes in the abdomen, palpation of spleen, liver and uterus; and incision of deep popliteal, subiliac, mammary, medial iliac, superficial cervical, caudal deep cervical, and prescapular lymph nodes in the carcass. Liver, lung, or other organ tissues were only submitted for further diagnostics if gross lesions were observed in the tissue. Tissue samples were submitted to the National Veterinary Services Laboratory (Ames, IA) in formalin (fixed) and borate (fresh). Pathologists examined formalin fixed tissue for lesions consistent with bTB. If acid fast stained bacteria were observed within a granuloma lesion on histopathology, the fixed tissue was tested by conventional PCR for *Mycobacterium tuberculosis complex* IS6110 DNA (*M*. *tb complex*) [3]. Fresh tissue specimens were cultured to isolate *Mycobacterium bovis (M*. *bovis)*. All cattle necropsied for bTB testing were euthanized and disposed of through rendering, alkali digestion or burial.

#### White-tailed deer

Deer surveillance was primarily conducted between 48° 11_N and 49° 0_ N latitude and 94°56_W and 95°58_W longitude in northwest Minnesota. All deer collected in the surveillance effort were killed by gunshot or archery and were taken by a) hunters licensed with the State of Minnesota under the authority of the Commissioner of Natural Resources or b) USDA APHIS Wildlife Services (WS) staff contracted by the same Commissioner. USDA APHIS WS staff collected deer on public lands and on private lands with signed authorization from landowners. All deer collected were examined by Minnesota Department of Natural Resources (DNR) personnel trained to detect lesions suspicious of bTB. These lesions included any abnormally colored (e.g. brownish-tan) foci in lymph nodes (particularly head, lung, and mediastinal nodes), lungs, and body cavities (particularly thoracic cavity). Sampling procedures included a visual inspection of the chest cavity of the deer. All animals with suspicious lesions were confiscated (with or without internal organs) and submitted to the Minnesota Veterinary Diagnostic Laboratory (MVDL) for further diagnostic testing. In animals without suspicious lesions, three pairs of head lymph nodes (parotid, submandibular, and medial retropharyngeal) were removed and submitted to MVDL for further testing and the carcass released to the hunter.

Further testing at the MVDL included visual inspection of the lymph nodes by supervised technicians and lab personal for the presence of gross lesions. If gross lesions were detected in the lymph nodes, histopathology of the lesion using routine hematoxylin and eosin stains and routine acid fast stain was performed. Fresh frozen and paraffin embedded fixed tissue from the individual animal were submitted to the NVSL for culture and testing for *M*. *tb complex* and *M*. *avium* by PCR as described for cattle [3]. If no gross lesions were detected by MVDL staff, tissue from five individual animals were pooled and sent to NVSL for culture. Formalin-fixed and unfixed lymph node samples from each individual animal were saved at MVDL for re-culture in case a pool was positive for bTB by culture at NVSL.

Deer with visible lesions submitted to the MVDL were disposed of via alkali digestion. In cases of hunter-harvested deer that cultured positive for bTB but no visible lesions were detected at the time of sampling, individuals were contacted regarding test results and could voluntarily surrender venison for alkali digestion; the remainder of those carcasses were placed in a landfill.

### Mycobacterial Cultures—Genotyping and Phylogenetic Analysis

Tissues were trimmed, placed in 0.06% sodium hypochlorite for 15 minutes, homogenated with phenol red broth and then decontaminated for 7–10 minutes using sodium hydroxide as described previously [4]. Tissue sediments were inoculated into both liquid media, BACTEC MGIT and BACTEC 12b, (Beckton Dickenson, Sparks, MD, USA) and solid media (in house Middlebrooks 7H11 supplemented with hemolyzed red blood cells, calf serum pyruvate and malachite green) and incubated for 6 weeks and 8 weeks respectively. Resulting isolates were confirmed as *M*.*tb* complex with commercial DNA probes (AccuProbe, Hologic, San Diego, CA, USA). Species determination and genotyping was initially conducted using spoligotyping and variable number tandem repeats (VNTR). [5, 6]. Spoligotyping was conducted using a Southern blotting technique where 43 non-repetitive spacer regions were amplified by PCR and the resulting products hybridized on to a prepared membrane VNTR was conducted by amplifying 8 by using fluorescently tagged PCR primers and then identified using fragment analysis with an ABI 3500XL instrument (Thermo Fisher Scientific, San Diego, CA, USA) [7].

To obtain the whole genome sequences, isolates associated with this outbreak were sequenced on a MiSeq instrument (Illumina, San Diego, CA) using 2x250 paired-end chemistry and the Nextera XT library preparation kit (Illumina, San Diego, CA). FASTQ files from the instrument were put through the NVSL in-house pipeline (see https://github.com/USDA-VS). Briefly, reads were aligned to the reference genome AF2122/97, NCBI accession number NC_0002945, using BWA and Samtools [8, 9]. A depth of coverage of 100X was targeted. BAM files were processed using Genome Analysis Toolkit (GATK)’s best practice workflow. SNP were called using GATK’s UnifiedGenotyper outputting them to variant call files (VCF)[10–12]. Results were filtered using a minimum QUAL score of 150 and AC = 2. From the VCFs, SNPs gathered were outputted to three formats: an aligned FASTA file; tab-delimited files sorted by position location and by SNP groups; and a maximum likelihood phylogenetic tree created with RAxML [13]. The tree was built using a GTR-CAT model with input taken as an alignment file containing only informative and validated SNPs. SNPs were visually validated using Integrative Genomics Viewer (IGV) [14]. FASTQ files from the isolates sequenced were uploaded into NCBI short read archive. Accession numbers are listed in the S2 Table.

Isolates were further characterized using molecular clock phylogenetic analysis to estimate times to introduction and transmission of bTB within the outbreak [15]. The time of most recent common ancestor (MRCA) and phylogeny of introduction of *M*. *bovis* was estimated with a Bayesian analysis using BEAST v.2.1.3 [16]. Tip dates for phylogenetic trees were defined as the year that the *M*. *bovis* isolates were sampled. A GTR+Γ substitution model was used. All permutations of a strict or relaxed molecular clock (uncorrelated log-normal distribution) and constant population size or Bayesian skyline [15] demographic models were evaluated using Akaike’s information criteria through Markov chain Monte Carlo (AICM; [17]; S2 Text). Based on observed SNP change rates of a *M*. *bovis* isolated from outbreaks throughout the United States (unpublished data), an informative lognormal prior was used for the molecular clock rate (untransformed mean = 0.005, untransformed sd = 0.3). All other priors were the default priors (https://code.google.com/p/beast-mcmc/wiki/ParameterPriors).

All permutations of the molecular clock models and demographic models were fit for the Minnesota isolates in addition to 6 sequenced isolates recovered from a herd in Texas, which were the most closely related isolates (based on SNP identity) in the NVSL archive to estimate phylogenetic relationship and time of divergence. The same analysis was performed for only the Minnesota isolates to infer the phylogenetic relationships, divergence timing, and demographic reconstruction of the well-sampled Minnesota outbreak. Model comparison methods are presented in S2 Text. Five chains of 100 million MCMC generations of the best fit model (molecular clock and demographic model) were run to ensure convergence. Point estimates and 95% Highest-Posterior Density (HPD) intervals for the time of MRCA and phylogenetic relationships on Minnesota isolates were obtained by sampling every 1,000^th^ generation of the combined posterior parameter distributions of the 5 chains after a 10% burn-in (total 450 million generations after burn-in). The Tracer v1.6 program [18] was used to visually evaluate convergence and generate parameter estimates.

A maximum clade credibility tree was created with median and 95% HPD heights (in years) for nodes with greater than 0.40 posterior probability using Fig Tree v1.4.2 [19]. Pairwise genetic distances between tips were calculated from this tree using the sum of branch lengths between tips. For cattle herds with more than one isolate, the tip with the shortest branch length to the tree root was used to represent the isolates from that herd. The pairwise genetic distances were compared against pairwise geographic distances and Pearson’s correlation coefficients were calculated among genetic and geographic distance of cattle herd pairs, deer pairs, and herd-deer pairs.

### Indemnification and Slaughter of Infected Cattle Herds

If evidence of *M*. *bovis* was confirmed at the NVSL, federal and state animal health officials determined whether the herd would be declared ‘Affected’ [20]. Indemnity with federal funds was offered for the affected herds and the animals were taken to slaughter with enhanced inspection.

### Statewide Surveillance

In addition to local surveillance, statewide surveillance for bTB in both cattle and deer was conducted to meet USDA requirements for the state’s cattle TB status. All cattle 12 months of age and older in a herd (a whole herd test) were tested using the CFT test. Herds were tested from a random selection of cattle herds throughout Minnesota outside of the TB affected area between June 2006 and December 2007. Samples collected from hunter harvested deer collected outside of the TB affected area during the 2007 fall hunting season were tested for bTB.

## Results

**Fig 1:**
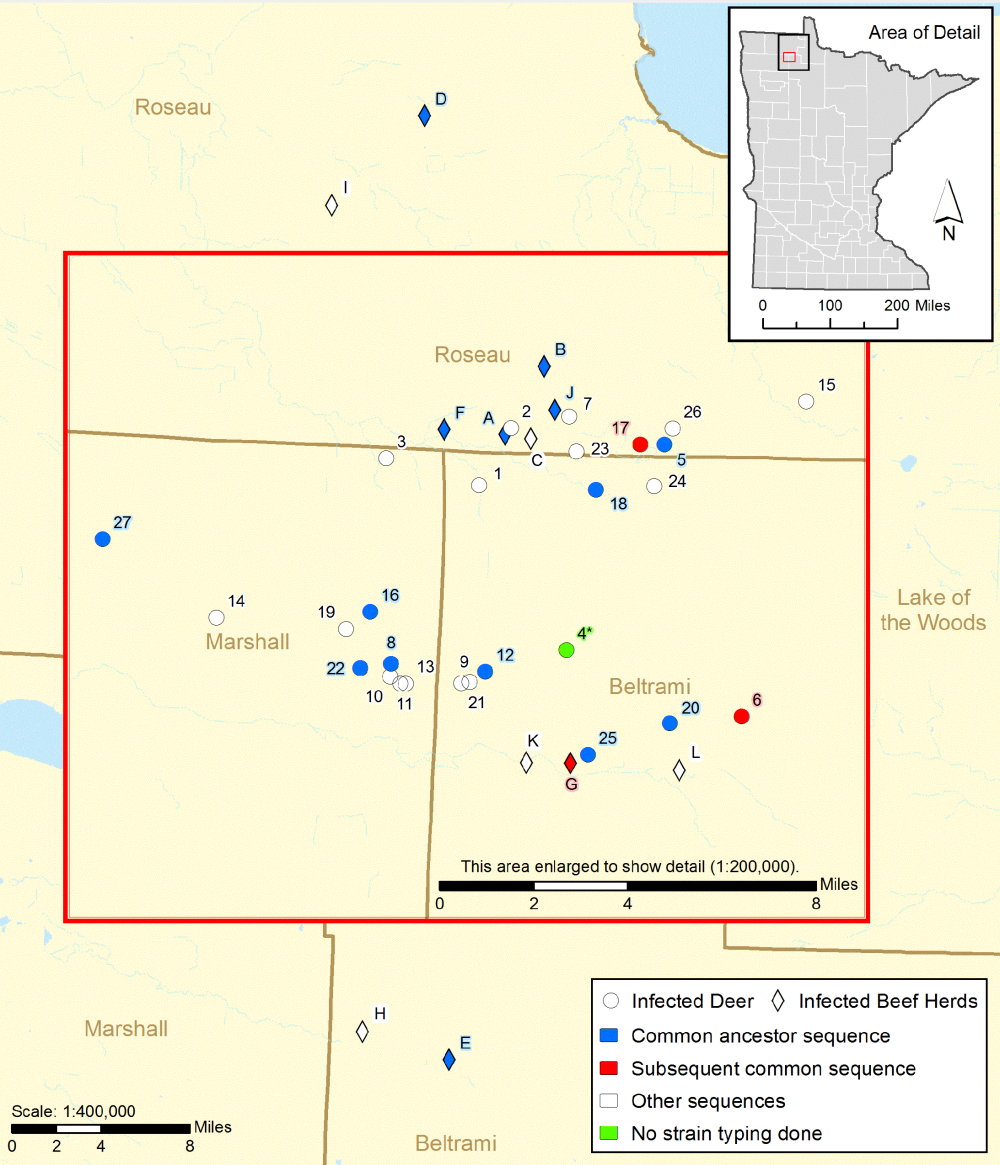
Locations of bTB Infected Beef Herds and White-tailed Deer.

A five year old beef cow was identified with lesions suspect bTB lesions at a slaughter plant in Wisconsin in February 2005. The subsequent epidemiological investigation identified twelve bTB infected beef cattle herds between July 2005 and January 2009, all within 43 miles of each other in Beltrami, Marshall, and Roseau Counties in northwestern Minnesota (Fig 1), (S1 Table). A total of 382 potential source herds and 925 potentially exposed herds were traced to eight states; Iowa, Texas, Montana, South Dakota, North Dakota, Nebraska, Wisconsin, and Kansas during this investigation. Of the twelve bTB positive herds identified, two were found at slaughter inspection (Herd A and Herd L). Six herds were found through tracing cattle movement in and out of infected herds. In only one of these herds (Herd D) were exposed animals found infected with bTB; the other five herds were tested and found to be infected. Four of the twelve herds were identified by area testing requirements as outlined in the TB UMR [1] (Table 1, S1 Text).

**Table 1:** Results of Whole Herd Tests from Infected Herds.

Herd	CFT test results^a^	Suspects / Reactors	# Animals found infected^b^
**A**	63/586	8 / 13	6
**B**	5/136	1 / 2	3
**C**	3/164	1 / 0	1
**D**	No herd test		
**E**	18/298	2 / 2	1 Reactor
**F**	1/15^c^	0 / 1	1
**G**	2/37	1 / 1	1 Reactor
**H**	2/57^c^	0 / 2	2
**I**	6/253^d^	0 / 1	1
**J**	2/62^d^	0 / 2	2
**K**	3/64^c^	0 / 1	1
**L**	No positive herd test		
12	105 / 1672	13 / 25	19

^a^ Number of responders (suspects) / number of animals tested in a whole herd test of all animals 12 months of age and older

^b^ Number of animals found infected in the whole herd test. Infection determined by *M*. *tuberculosis complex* PCR positive on fixed tissue and / or culture of *M*. *bovis* or *M*. *tuberculosis complex* from tissue

^c^ Results from the second annual whole herd test

^d^ Results from the third annual whole herd test

Surveillance in free-ranging white-tailed deer was initiated in late 2005 and occurred over eight years through the 2012 hunting season with 10,667 animals tested for bTB. The majority of the sampling was conducted through hunting (7,839 deer); however agency sponsored sharpshooting conducted in the winters of 2007–2010 in key areas where previously infected deer were found accounted for an additional 2,613 deer tested. Landowner shooting permits collected 215 deer. In total, 27 TB positive deer were identified, all within a 10 mile radius of the index cattle farm (S1 Text).

### Diagnostic Results

#### Cattle

**Fig 2:**
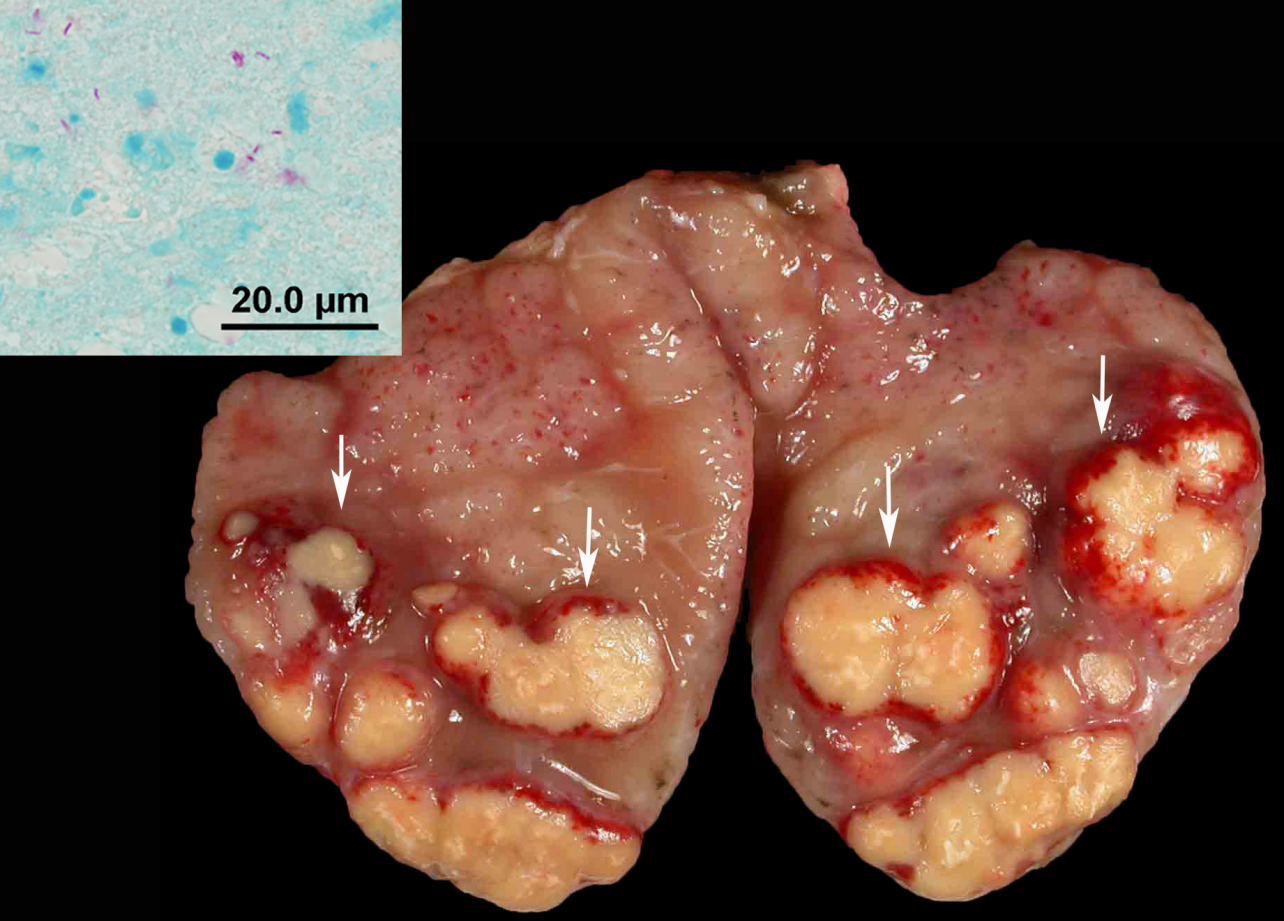
Cut section of a tracheo-bronchial lymph node from a cow in Herd A. The node was edematous and contained multiple well demarcated beige mycobacterial granulomas bordered by a red rim (arrows). Inset: Photomicrograph of gross lesion with rare acid-fast-positive bacilli. Modified Ziehl-Neelsen stain.

Thirty-seven cattle from the 12 infected herds were found infected with *M*. *bovis*. All infected cattle had visible gross lesions, primarily found in head and thoracic lymph nodes. Of the 37 infected cattle, eight had gross lesions of bTB found only in head lymph nodes and 19 animals had gross lesions found only in thoracic lymph nodes. In two animals, gross lesions were found only in the abdominal lymph nodes. Five animals had lymph node lesions found in more than one area of the body—head, thorax, or abdomen, and one had lesions in a prescapular lymph node. Nodular or granuloma lesions were found infrequently in organ tissues; one animal has lesions in the lung and spleen and two different animals had lesions found in the liver (Table 2). The location of lesions of one animal identified through slaughter inspection in another state was not available. Lymph node lesions varied from single to multifocal and in consistency from yellow caseous to granular to calcified and in size from less than 1/8 inch to greater than 6 inches in diameter (Fig 2).

**Table 2:** Isolate ID, Sex, Age, and Diagnostic Findings for each Infected Animal.

Isolate ID	Sex^a^	Age (years)	Specimen (Herd Letteror Deer Number)	Infected tissues^b^	Gross lesions	AFS^c^ positive	FFPE MTBC PCR^d^	*M*. *bovis* culture positive
05–4040	F	5	Carcass (Herd A)	Unidentified lymph node	Yes	Yes	Yes	Yes
05–8171	F	3	Carcass (Herd A)	Thoracic lymph nodes	Yes	Yes	Yes	Yes
No isolate	F	5	Carcass (Herd A)	Head, tracheobronchial (TrB) lymph nodes	Yes	Yes	Yes	No
	F	5	Carcass (Herd A)	Head and thoracic lymph nodes	Yes	Yes	No	Yes
No isolate	F	6	Carcass (Herd A)	TrB lymph node	Yes	Yes	Yes	No
	F	8	Carcass (Herd A)	Retropharyngeal (RP), prescapular lymph nodes	Yes	No	Not tested	*M*. *tb complex*
	F	10	Carcass (Herd A)	Head lymph node	Yes	Yes	Yes	Yes
05–9943,9945	M	5	Carcass (Herd B)	Head, abdominal lymph nodes	Yes	Yes	Yes	Yes
05–9941,9942	F	14	Carcass (Herd B)	Head lymph node	Yes	Yes	Yes	Yes
05–9937	F	14	Carcass (Herd B)	Thoracic lymph node	Yes	Yes	No	Yes
05–9955, 9963	F	1.5	Carcass (Herd C)	Head lymph node	Yes	Yes	Yes	Yes
	F	3	Carcass (Herd D)	Unidentified lymph node	Yes	Yes	No	Yes
	F	3	Carcass (Herd D)	Unidentified lymph node	Yes	Yes	No	Yes
05–10941	F	3	Carcass (Herd D)	Unidentified lymph node	Yes	Yes	Yes	Yes
06–1531,1534	F	10	Carcass (Herd E)	Head lymph node	Yes	Yes	Yes	Yes
06–3322	F	0.75	Carcass (Herd E)	Mesenteric lymph node	Yes	Yes	Yes	Yes
06–3345	F	0.75	Carcass (Herd E)	Mediastinal lymph node	Yes	Yes	Yes	Yes
06–10979,84	F	2	Carcass (Herd F)	Head lymph nodes, lung, spleen	Yes	Yes	Yes	Yes
07–0608	F	2	Carcass (Herd G)	RP lymph node	Yes	Yes	Yes	Yes
08–0323	F	1.5	Carcass (Herd H)	RP, thoracic lymph nodes, liver and Peyer’s Patches lesions	Yes	Yes	Yes	Yes
08–3591	M	1.5	Carcass (Herd H)	Bronchial lymph node	Yes	Yes	Yes	Yes
08–1897	F	5	Carcass (Herd I)	Mediastinal lymph node	Yes	Yes	No	Yes
08–2634	F	10	Carcass (Herd J)	TrB, mediastinal lymph nodes	Yes	Yes	Yes	Yes
08–2629, 2630	F	12	Carcass (Herd J)	Mediastinal and mesenteric lymph nodes	Yes	Yes	Yes	Yes
08–1568	F	8	Carcass (Herd K)	Head and TrB lymph nodes	Yes	Yes	Yes	Yes
09–0573	F	13	Carcass (Herd L)	Mediastinal lymph node	Yes	Yes	Yes	Yes
09–0615	F	7	Carcass (Herd L)	Mediastinal lymph node, liver	Yes	Yes	Yes	Yes
09–0625	F	5	Carcass (Herd L)	Mediastinal lymph node, spleen	Yes	Yes	Yes	Yes
	F	1	Carcass (Herd L)	Mediastinal lymph node	Yes	Yes	Yes	Yes
09–3403	MC	1	Carcass (Herd L)	Mediastinal lymph node	Yes	Yes	Yes	Yes
09–3410	MC	1	Carcass (Herd L)	Mediastinal lymph node	Yes	Yes	Not tested	Yes
09–3411	F	1	Carcass (Herd L)	TrB lymph node	Yes	Yes	Yes	Yes
09–3405	MC	1	Carcass (Herd L)	TrB lymph node	Yes	Yes	Not tested	Yes
09–3404	F	1	Carcass (Herd L)	Mediastinal lymph node	Yes	Yes	Not tested	Yes
09–3407	F	1	Carcass (Herd L)	RP lymph node	Yes	Yes	Not tested	Yes
09–3409	MC	1	Carcass (Herd L)	Mediastinal lymph node	Yes	Yes	Not tested	Yes
	MC	1	Carcass (Herd L)	Mediastinal lymph node	Yes	No	Not tested	Yes
06–1254	M	6.5	FD carcass^e^ (Deer #1)	Head lymph nodes	Yes	Yes	Yes	Yes
06–3639, 3641	F	5.5	Carcass (Deer #2)	Lungs and pleura	Yes	Yes	Yes	Yes
07–1287	M	≥2.5	FD carcass (Deer #3)	Head lymph node	Yes	Yes	Yes	Yes
	M	≥2.5	FD carcass (Deer #4)	Head lymph node	Yes	Yes	Yes	Yes
07–1366	M	≥2.5	FD carcass (Deer #5)	Head lymph node	Yes	Yes	Yes	Yes
07–3280	F	≥2.5	Head lymph nodes (Deer #6)	Head lymph node	Yes	Yes	Yes	Yes
07–2555	M	2.5	Head lymph nodes, lungs (Deer #7)	Lungs and pleura	Yes	Yes	Yes	Yes
07–5776,5777	F	6.5	Carcass (Deer #8)	Lungs, pleura, and omentum	Yes	Yes	Yes	Yes
07–6071	F	1.5	Head lymph nodes, lungs (Deer #9)	Head lymph node	Yes	Yes	Yes	Yes
07–6922	F	2.5	Carcass (Deer #10)	Lungs and pleura	Yes	Yes	No	Yes
	F	3.5	Carcass (Deer #11)	Lungs, pleura, and peritoneum	Yes	Yes	No	Yes
07–7693	M	1.5	Carcass (Deer #12)	Lungs and pleura	Yes	Yes	Yes	Yes
	F	3.5	Carcass (Deer #13)	Lungs and pleura	Yes	Yes	Yes	Yes
08–1772, 3040	M	≥2.5	Head lymph nodes (Deer #14)	Head lymph node	Yes	Yes	Yes	Yes
08–0863	M	4.5	FD carcass (Deer #15)	Lungs and pleura	Yes	Yes	Not tested	Yes
08–1624	M	≥2.5	Head lymph nodes (Deer #16)	Head lymph nodes	No	Yes	Yes	Yes
08–0864	M	4.5	FD carcass (Deer #17)	Head lymph nodes and lungs	Yes	Yes	Not tested	Yes
08–1021	M	≥2.5	Head lymph nodes, internal organs (Deer #18)	Head lymph node	Yes	Yes	Not tested	Yes
08–5416	F	≥2.5	Head lymph nodes (Deer #19)	Head lymph node	Yes	Yes	No	Yes
08–4363	M	3.5	Head lymph nodes, lungs (Deer #20)	Lungs and head lymph node	Yes	Yes	Yes	Yes
08–4364	M	4.5	Carcass (Deer #21)	Lungs and mesenteric lymph node	Yes	Yes	Yes	Yes
08–4570	M	6.5	Carcass (Deer #22)	Lungs, pleura, brain, and RP lymph node	Yes	Yes	Yes	Yes
08–4576	F	6.5	Carcass (Deer #23)	Lungs, pleura and retropharyngeal lymph node	Yes	Yes	Yes	Yes
08–4741, 4741	F	3.5	Carcass (Deer #24)	Lungs, pleura, omentum, and RP lymph node	Yes	Yes	Yes	Yes
09–5373, 6718	F	7.5	Head lymph nodes (Deer #25)	Head lymph node	No	Yes	No	Yes
09–4591, 4592	M	6.5	FD carcass (Deer #26)	Lungs, pleura, trachea, and heart	Yes	Yes	No	Yes
10–1090	M	3.5	Head lymph nodes (Deer #27)	Head lymph node	Yes	Yes	Yes	Yes

^a^ F = female; M = male; MC = steer

^**b**^ Tissues identified as having indications of infection with Mycobacterium–PCR or culture positive.

^**c**^ AFS = Acid fast staining

^d^ *Mycobacterium tuberculosis complex* PCR performed on the formalin fixed paraffin embedded tissue where acid fast organisms were identified.

^e^ FD = field dressed carcass: the internal organs were removed in the field by hunters and not available for examination by pathologists. The head was also removed in the field and not available for examination by pathologists. However head lymph nodes were removed and submitted along with the field dressed carcass.

Acid fast staining bacteria were identified in fixed tissues in all but two of 35 animals evaluated; one of these animals cultured positive for *M*. *tb complex* and the other for *M*. *bovis*. Two animals with acid fast staining bacteria identified in fixed tissues did not culture positive for *M*. *bovis* but were positive for *M*. *tb complex* by PCR on fixed tissue (Table 2). Twenty-five of 30 cattle tested positive for *M*. *tb complex* PCR performed on formalin fixed paraffin embedded tissue. Cattle were not tested if they were negative for acid fast staining bacteria or suspect animals in Herd L were not all tested due to the high number of lesioned animals found in the herd. *M*. *bovis* was isolated from 34 of 37 cattle; *M*. *tb complex* was identified from one additional animal. The two animals with no isolate of bTB bacteria were positive by *M*. *tb complex* PCR (Table 2).

Other mycobacteria were isolated from necropsied animals including *M*. *smegmatis*, *M*. *normochromogenicum*, *M*. *avium*, and others. Coinfections of M. bovis and M. smegmatis were identified in two animals.

#### White-tailed deer

**Fig 3:**
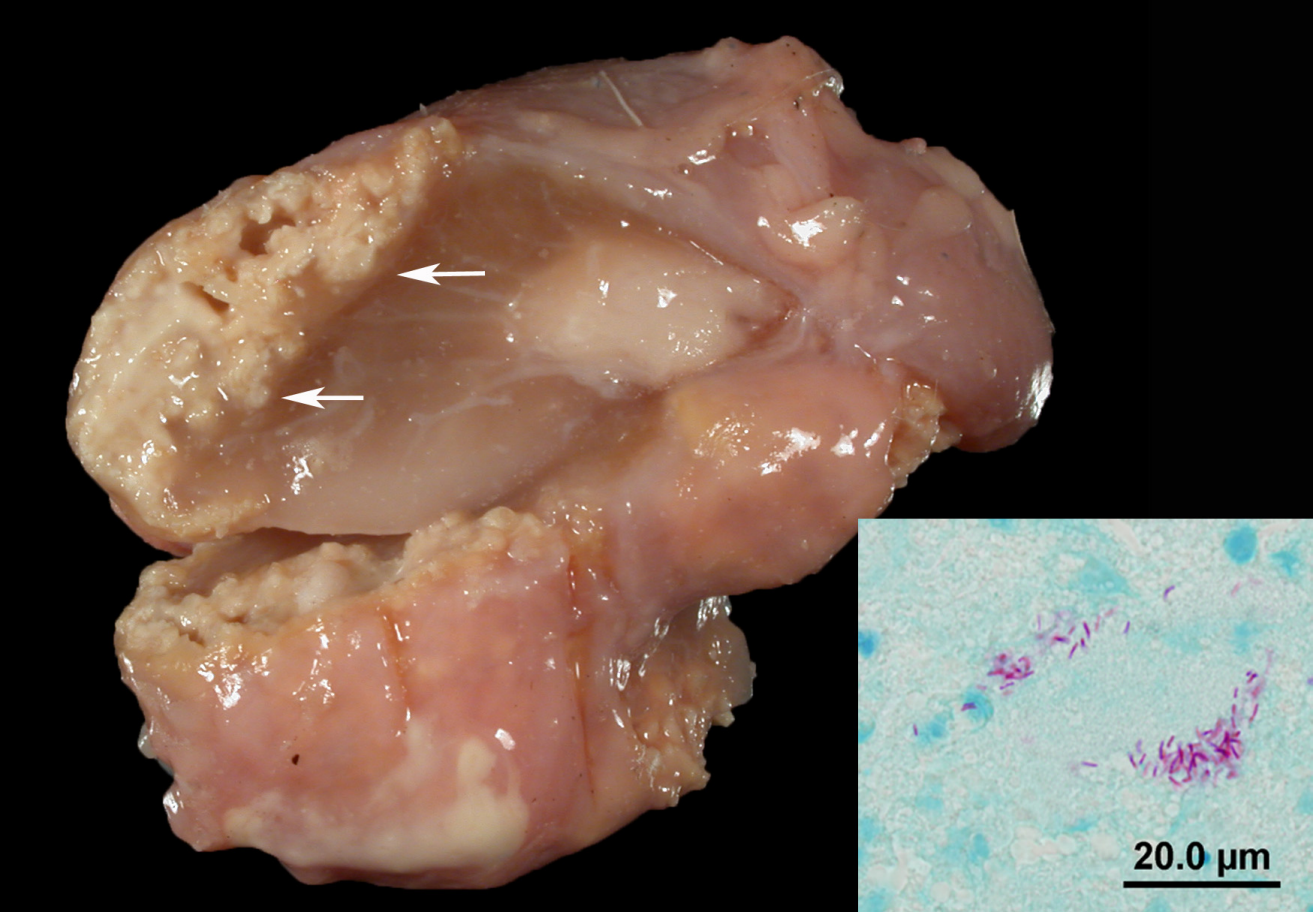
Cut section of the retropharyngeal lymph node from Deer #4. A well demarcated beige mycobacterial granuloma with calcification is present in the periphery of the node (arrows). Inset: Photomicrograph of gross lesion with rare acid-fast-positive bacilli. Modified Ziehl-Neelsen stain.

**Fig 4:**
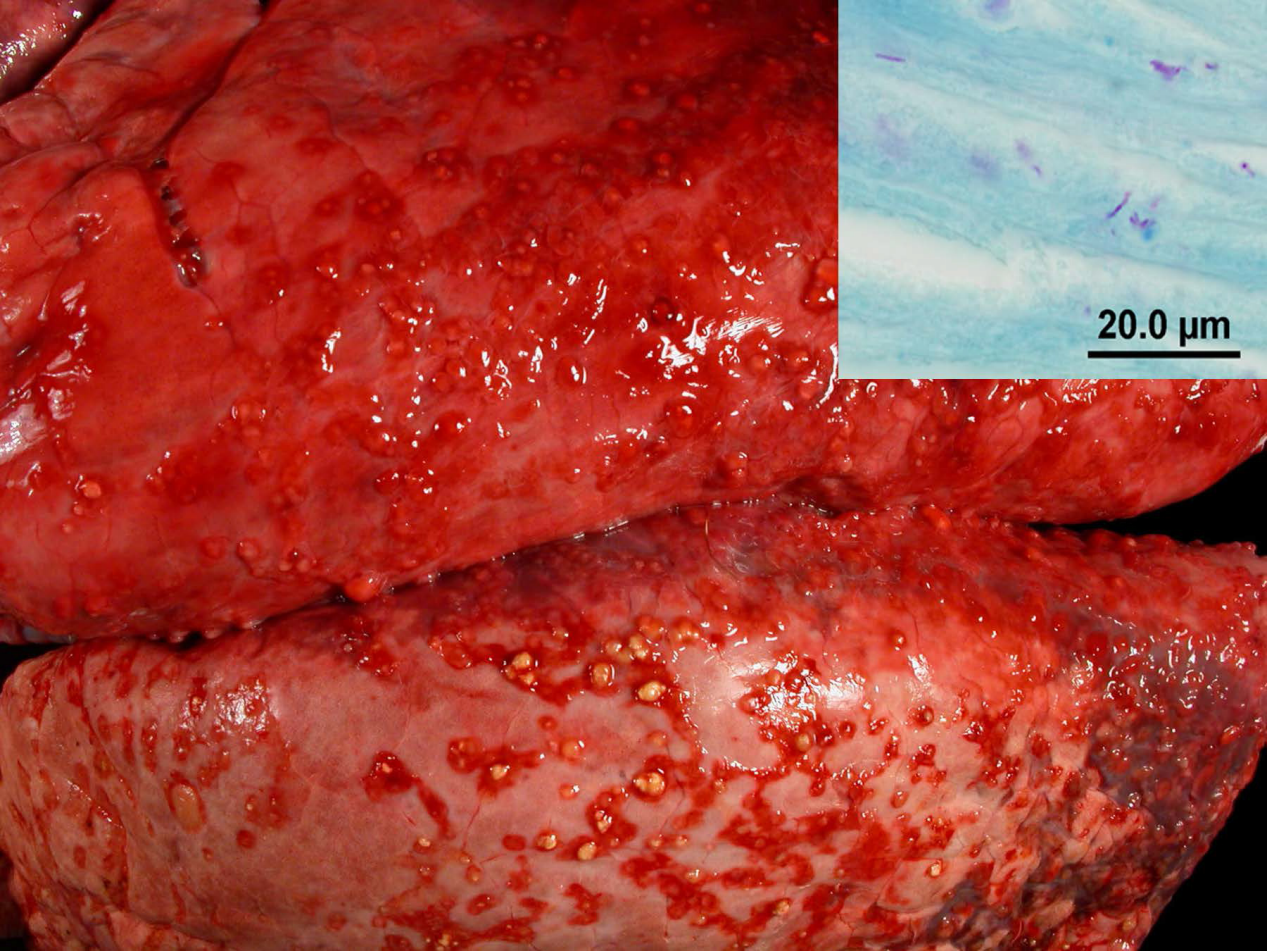
Lungs of Deer #2: Numerous mycobacterial granulomas are present on the pleura. Inset: Photomicrograph of gross lesion with rare acid-fast-positive bacilli. Modified Ziehl-Neelsen stain.

The 27 deer confirmed with bTB were all diagnosed with a positive *M*. *bovis* culture; 19 of 24 tested for *M*. *tuberculosis complex* by PCR on formalin fixed paraffin embedded tissues were positive (Table 2). Lesions were primarily found in the lymphatic and respiratory systems. Sixteen animals were eviscerated carcasses with suspicious lesions identified by DNR field personal; 12 of these animals had lungs submitted with the carcass. In 11 of these 12 carcasses, abdominal organs were also included among the submitted specimens. The remaining 11 of 27 animals included four animals in which head lymph nodes and the pluck were submitted, and seven animals in which only head lymph nodes were submitted (Table 2). Head lymph node lesions, detected in 18 deer, ranged from focal small largely calcified granulomas, to more florid (less calcified) granulomas to abscess-like lesions (Fig 3). Of the submitted carcasses (n = 16), all animals were in a good nutritional state. In all but 3 of the 16 carcasses, the bTB lesions were limited to the head lymph nodes, pleura, and/or lungs. In the 16 animals, in which lungs and head lymph nodes were available for examination, 5 animals had both granulomatous lymphadenitis and pleuritis/pleuropneumonia while 11 animals had granulomatous pleuritis/ pleuropneumonia but no lesions in the head lymph nodes. In the 16 animals with intrathoracic lesions, the intrathoracic lesions were limited to the mesothelial lining in six animals. These animals had multifocal to coalescing granulomatous pleuritis and in one case also a multifocal to coalescing granulomatous epicarditis and tracheitis. Of the 16 animals with intrathoracic lesions, ten animals had lung involvement besides the pleural involvement. The lung lesions consisted of small multifocal florid (less calcified) granulomas in 9 animals (multifocal to coalescing granulomatous pleuropneumonia) while one animal had large areas of pyogranulomatous inflammation (Fig 4). In 3 animals, granulomatous or calcified lesions were detected in the peritoneal lining, omentum, and rare mesenteric lymph nodes. These 3 deer also had granulomatous pleuritis/pleuropneumonia.

In 25/27 of the deer cases, gross lesions were detected by field personnel or the repeat gross examination at MVDL. In two of the above mentioned cases, in which only lymph nodes were submitted, gross lesions were not detected but the culture of the pooled lymph nodes was positive for *M*. *bovis*. In these two cases, the stored fixed and unfixed samples from the individual animals were retrieved and re-examined. In both pools, a fixed lymph node sample was identified with a small calcified lesion that was initially missed and reculture attempts confirmed a positive case among the five pool members in each pool. A variety of non-tuberculoid *Mycobacterium sp*. were isolated from other lymph node pools from deer. Most of these bacteria were not further identified. Few belonged to the *M*. *terrae* complex and *M*. *avium* complex. *M*. *smegmatis* was isolated from a deer with pleuritis.

### Mycobacterial Culture Results—Genotyping and Phylogenetic Analysis

All isolates from cattle and deer found during this outbreak were indistinguishable with each other using spoligotyping and VNTR. The spoligotype, SB0271 (Octal code: 664073377777600) had not previously been identified in USA origin cattle or from cattle originating from Mexico that were slaughtered in the USA. However, the parent spoligotype SB0140, differing by only one spacer region, was commonly found in Mexican origin imports.

In total, 65 isolates were sequenced from this outbreak (34 cattle, 31 deer; some individuals had multiple isolates from separate *M*. *bovis* lesions). Ten cattle from six herds, Herds A, B, D, E, F, and J, and eleven deer had the same WGS SNP profile, which was the MRCA for all the isolates in the outbreak. (S2 Table). Detection of animals with the MRCA varied both temporally (from 2005 to 2009) and spatially (Fig 1). The remainder of isolates showed genetic divergence both between and within herds and animals, indicating subsequent transmission events after bTB was introduced into Minnesota. Subsets of animals were found with common sequences indicating relatedness between those animals. To examine repeatability of sequencing and genotypes within the animals, nineteen isolates recovered from 2 different sites within the same animal from 10 animals, 4 deer and 6 cattle, were sequenced. Eight of the animals had no detectable SNP differences between each site. Two animals however, Deer 24, and the cow labeled Herd E-1 had minor differences. This is consistent with a report in experimental infection and suggests care needs to be taken to characterize the population of bacteria within the animal [21]. The S2 Table contains parsimonious informative SNPS including position information in relation to the reference of the isolates.

**Fig 5:**
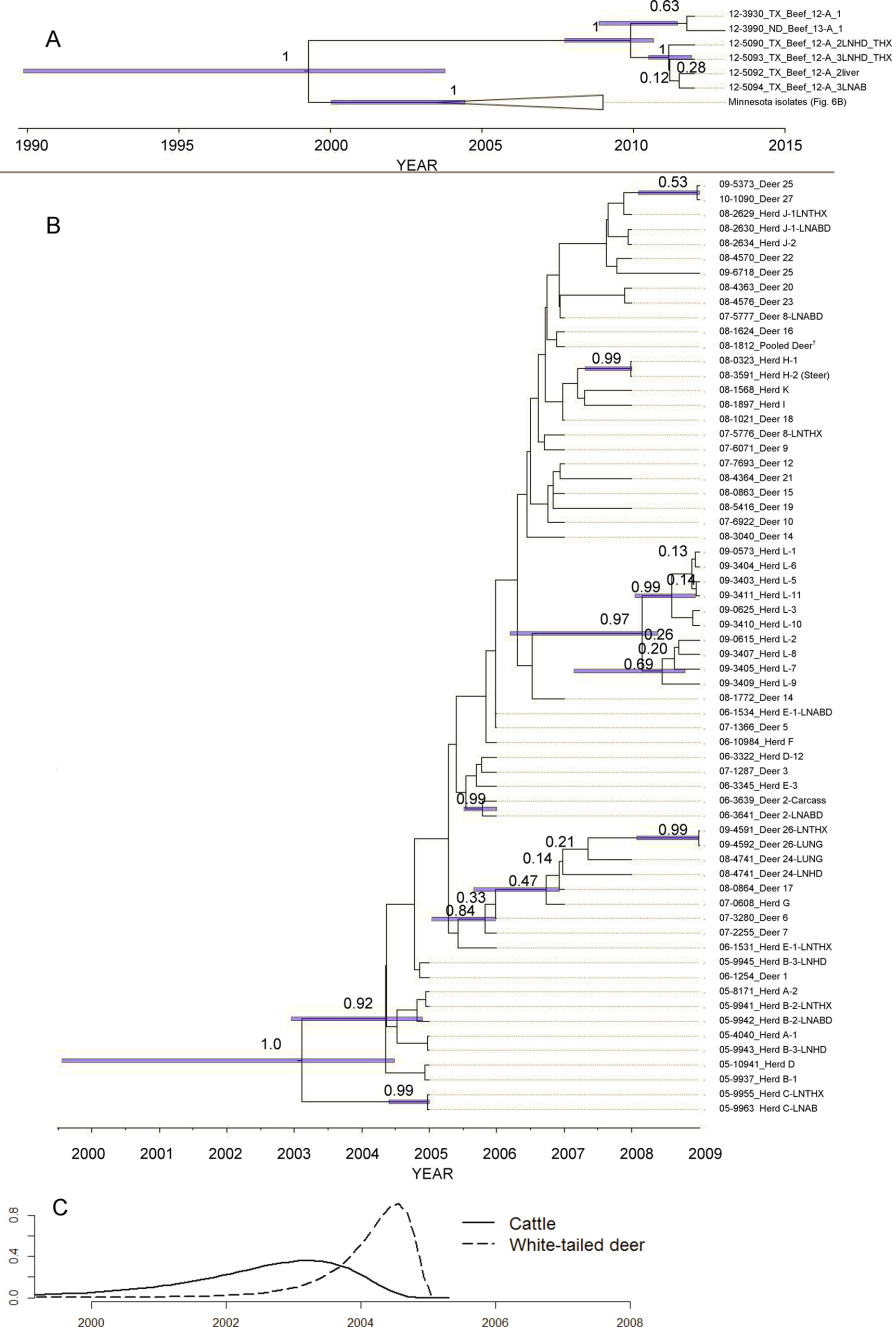
Estimated time of most recent common ancestor (MRCA) of *Mycobacterium bovis* and Bayesian maximum clade credibility phylogeny from affected cattle farms and wild white-tailed deer in Minnesota, 2005–2009, and *M*. *bovis* isolates associated with a Texas beef herd sampled 2013–2014. (A) Phylogenetic relationship between the Minnesota and Texas isolates with an estimated MRCA of 1999 (95% HPD [1991, 2005]). Node labels are the posterior probability. 95% HPD bars are displayed on nodes with >0.4 posterior probability (B) Isolates from only the Minnesota phylogeny in an unresolved maximum clade credibility tree. Nodes with ≤ 0.40 posterior support are collapsed onto unresolved branches. Node labels are the posterior probability and 95% HPD bars are displayed on nodes with >0.4 posterior probability (C) Density of marginal posterior probability MRCA estimates for *M*. *bovis* isolated from cattle and white-tailed deer from the Minnesota outbreak. The isolate names contain information on species (Deer = white-tailed deer and Herd = cattle), cattle herd (A-L; S1 Text), and individual deer identification (1–27); corresponding to the S2 Table. White-tailed deer isolate marked †, was from a pooled sample from multiple individuals.

The Bayesian molecular clock phylogenetic analysis and the maximum likelihood phylogenetic analysis of the isolates indicated that the *M*. *bovis* isolates from cattle and white-tailed deer in Minnesota were monophyletic. The *M*. *bovis* isolates from the Minnesota outbreak were distantly related to the most closely related *M*. *bovis* isolates from a Texas beef herd detected in 2012 with a most likely time to MRCA in the 1990’s (median = 1999, 95% HPD interval [1991, 2005]; Fig 5A; S3 Table).

**Fig 6:**
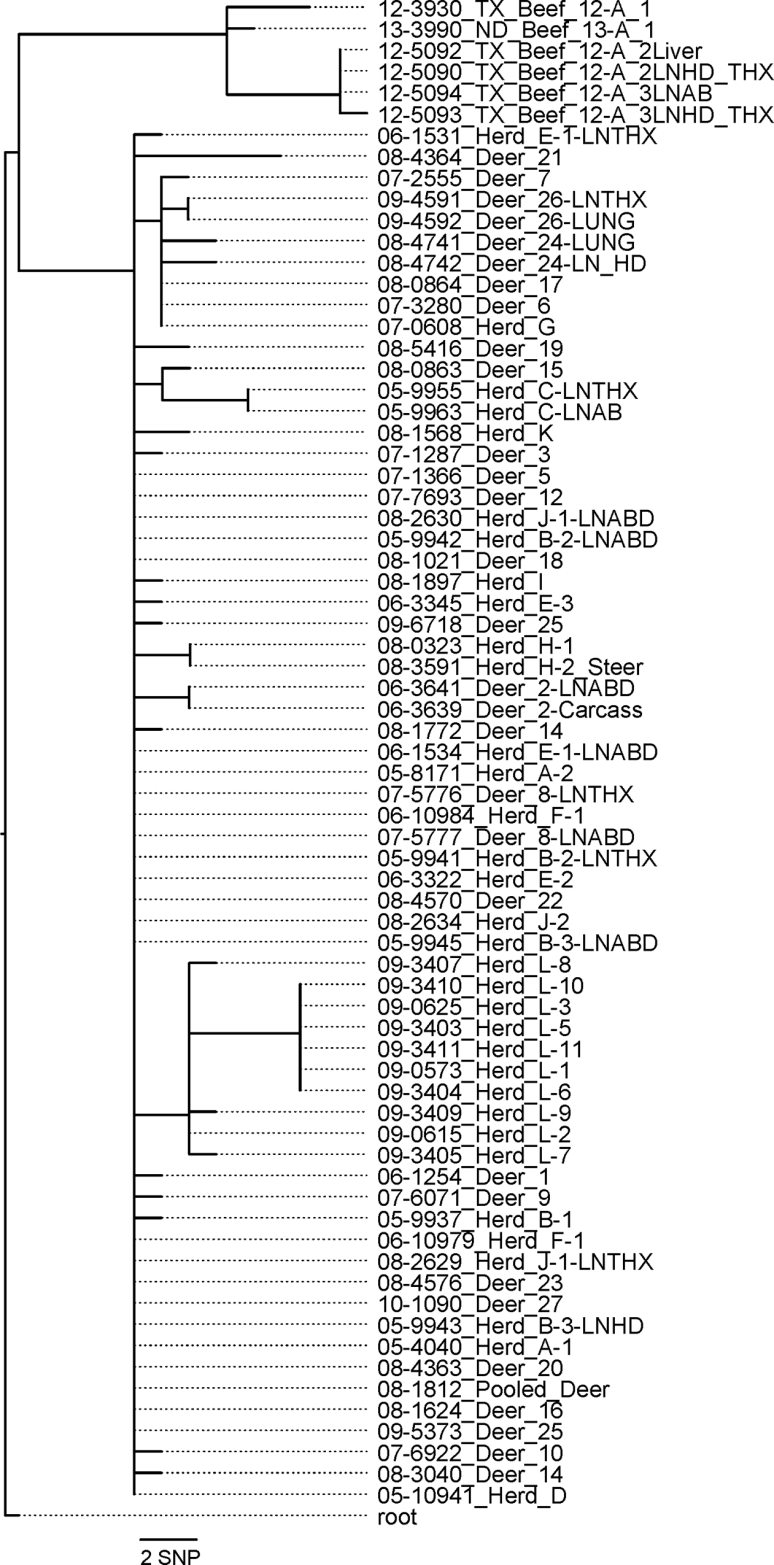
RAxML phylogenetic tree built using GTR-CAT model using an alignment containing only informative, validated SNPs. The tree contains all sequenced isolates recovered from the Minnesota outbreak and the most closely related isolates sequenced by the NVSL which were recovered from a cattle herd in Texas.

The maximum likelihood analysis indicated that the most frequent SNP sequence isolated was also the common ancestor sequence with all subsequent SNP changes diverging from this most frequent sequence (Fig 6). The Bayesian molecular clock phylogenetic analysis of the *M*. *bovis* isolates from cattle and white-tailed deer in Minnesota had a MRCA age estimated at 6.6 years (95% HPD: [4.45, 9.41]) from the most recent isolate (median year = 2002; 95% HPD = [1999, 2004]). The median date of MRCA of isolates from cattle was 2002 (95% HPD = [1999, 2004]) and the median date of MRCA from white-tailed deer was 2004 (95% HPD = [2002, 2004]; Fig 5). There was strong evidence for two divergence events near the root of tree (posterior probabilities 1.0 and 0.92; Fig 5), as well as events that indicate a separate divergent genotype isolated from white-tailed deer and a single cattle in herd G (posterior probability = 0.84; Fig 5), isolates from cattle in herd L (posterior probability = 0.97; Fig 5), herd H (posterior probability = 0.99; Fig 5), and two whited-tailed deer sampled in 2009 (posterior probability = 0.53; Fig 5). Model selection results indicated that the best model fit to the data included a constant molecular clock rate across branches and taxa (mean = 4.99x10^-3^ SNP changes per site per time; 95% HPD = [2.32x10^-3^, 8.01x10^-3^]; S4 Table) and an increasing effective population size through the outbreak (S1 Fig). There was no evidence of strong correlation between pairwise genetic and geographic distances (Cattle herd-herd pairwise Pearson’s correlation coefficient = -0.19, deer-deer = 0.12, herd-deer = -0.12;).

### Findings in Enhanced Slaughter of Infected Cattle Herds

The first eleven herds were indemnified by USDA and slaughtered using an enhanced inspection procedure which condemned all animals with suspect bTB lesions. Although four animals in Herd A were condemned at slaughter for gross lesions consistent with bTB, no further testing was done and these animals are not included in the total count of infected animals in this outbreak. Follow up diagnostic testing on samples from the condemned animals was performed for Herds B through K and are included in the total number of infected animals identified in this outbreak. Herd L participated in a state voluntary buy-out program and was identified as infected with bTB when animals were shipped to slaughter and suspect lesions were identified in carcasses during inspection; this herd had tested negative in two previous annual whole herd tests.

### Statewide Surveillance Results

In addition, statewide surveillance testing for bTB tested another 1551 cattle herds and 4000 white-tailed deer with no bTB infection found in Minnesota.

## Discussion

One goal of the investigation was to identify the source or introduction of the infection into Minnesota. Strain typing the organism cultured from infected animals by spoligotyping was able to characterize the bacteria as most similar to bTB isolates from Mexico and differs from the strain found in cattle and deer in Michigan or cattle and elk in Manitoba, the geographically closest known endemic areas. Bayesian molecular clock analysis of the whole genome sequences indicated that a single recent introduction of bTB into Minnesota with subsequent divergence between and within cattle herds and white-tailed deer is the most likely source of the outbreak. The molecular clock analysis estimated that the common ancestor of all Minnesota *M*. *bovis* isolates existed between 1999 and 2004 (Fig 5A). This estimate of common ancestor age does not necessarily indicate when the introduction into Minnesota occurred, but places a bound on the earliest possible time of introduction. No evidence identifying a specific movement or specific point of disease introduction into Minnesota was found. The index herd (Herd A) had the most interstate movement of animals in and out of the herd of any of the twelve infected herds. Two of the infected animals identified in the Herd A had been imported from Iowa five years prior to finding the infection in the herd. Iowa animal health regulators tested the herd of origin of those animals and found no infection present in the Iowa herd. No other infected animals found in the other eleven herds had been imported from another state.

Six herds found infected in this investigation all had at least one negative herd test prior to finding an infected animal in the herd. Using the CFT test, a low sensitivity test, to detect a low prevalence disease in small herds likely contributed to the difficulty in detecting bTB [22]. In a risk assessment of bTB in Minnesota, epidemiologists from the USDA’s Center for Epidemiology and Animal Health evaluated bTB transmission in the first eleven cattle herds and the expected number of false negative herds given five known infected herds identified in 2005. They found that six herds, the number of herds identified as infected after one negative herd test in Minnesota, was twice the number of false negatives expected [23]. They theorized that active transmission was likely the cause for some of the herds testing positive after testing negative in previous years.

In 2012, three years after the last bTB infected deer was detected, a beef herd was found in Texas that was indistinguishable from the strain found in this outbreak using spoligotyping and VNTR. Despite a vigorous investigation, no epidemiological link to Minnesota was found. Once these Texas isolates were sequenced it became apparent they diverged prior to the common ancestor that was detected in Minnesota. The BEAST analysis suggests that the Minnesota outbreak and the Texas herd likely originated from a MRCA in the 1990’s. It is unknown if the MCRA existed in the USA or Mexico.

Connections between infected herds were identified in the investigation. Eight herds were found to have had cattle movement or fence line contact with other infected herds by tracing cattle movement records from the herds or local sales barn. Two other herds identified by area testing had no documented cattle movement or contact with the other ten herds found but had fence line contact between animals in their two herds. WGS of these isolates provided additional insight into the relatedness of the isolates and therefore the animals or herds (S2 Table). A maximum likelihood analysis of the SNP identities suggested that the common ancestor sequence was found from infected animals in six herds and eleven deer and was maintained through the remainder of the outbreak along with additional sequence mutations (Fig 6). In contrast, a Bayesian molecular clock analysis using the SNP sequences and date of isolate collection indicated that a common ancestor was not sampled (Fig 5). This discrepancy arises from an isolate from Herd C and one deer (deer 15) that shared a common mutation not found in any other isolates (T to C change at chrom-1-2353208; S2 Table). In addition to the common mutation shared between Herd C and deer 15, isolates from herd C had 3 additional unique mutations. When the dates of isolate collection and a molecular clock model were used, there was strong support (posterior node probability = 0.92; Fig 5B) for the divergence of the sequence in Herd C from an unobserved common ancestor, suggesting identity by state with the isolate from deer 15 at chrom-1-2353208. The alternative divergence from a common observed ancestor (identity by decent; Fig 6) was considered unlikely in the molecular clock analysis because the isolates from herd C had 3 additional SNP changes (three C to T changes; S2 Table), yet were collected early in the outbreak, making accumulating that many changes highly unlikely under the best fit constant molecular rate model. However it should not be overlooked that the collection date does not likely correlate to infection status of either active replication or latent state. Because of *M*. *bovis*’s latency and the relatively short time span of this outbreak using the collection date is likely a source of error in the Bayesian molecular clock analysis if mutation rates are different during active and latent states. We are not able to resolve this difference because we do not have the resolution of data or knowledge of factors that may drive different rates of change in *M*. *bovis* genetics. Nonetheless, this difference has minimal impact of the epidemiological conclusions: *M*. *bovis* was most likely introduced into Minnesota from a single source.

Cattle contact or movement was documented between many of these herds with the exception of Herd E. Herd E is located over 18 miles from the cluster of the other four herds with this sequence and over eight miles from the nearest infected deer collection site. Movement of people and vehicles between Herd E and Herd A were known to occur and transmission may have occurred through a fomite.

An animal in Herd G and two deer had the same single mutation from the initial sequence indicating a common source introduced infection among these animals. As Herd G had no known cattle movement with any other infected herd, the most likely explanation for this herd’s infection may be via transmission from deer. These infected deer were not collected on this farm but 4 miles and 6.5 miles from Herd G. Three additional deer had this single mutation plus additional mutations indicating transmission occurred from the strain identified in Herd G and the two deer to this next subset of animals. Given that infection in proximate cattle was not detected, despite intense surveillance, this suggests that transmission among white-tailed deer was occurring to propagate these mutations.

Isolates from Herd L contain several mutations within the herd. Two adults and four yearlings have the same four mutations from the common ancestor and a second group of animals, one adult and three yearlings, have the same two mutations and these three yearlings each have an additional mutation all different from each other. The two primary sets of mutations in this herd may be explained with the separation of this herd on pasture during summer grazing.

## Conclusions

The investigation and subsequent genomic sequencing and analysis of isolates from infected animals in this outbreak all point to a recent introduction of bTB into Minnesota and subsequent spread between cattle and deer in the area of the outbreak. Genomic sequencing clusters the isolates closely together based on the low number of mutations from the first strain introduced into Minnesota and given the few mutations that occurred, transmission had not been going on over a prolonged period of time. Additional evidence for this is in the age of infected deer identified, as there was an absence of younger animals found with the disease as the surveillance progressed. This further supports the disease was not established in deer; thus preventing the development of a reservoir [24]. The clustering of related divergent SNP changes within cattle herds (for example, herd L) indicates transmission within the herd without cross-species transmission. Also clustering of isolates with multiple divergent events in white-tailed deer isolates (isolates 6, 7, 17 24, 26), clustered with a single isolate from herd G, suggests that transmission occurred independently among white-tailed deer in this clade. Spatially, most of the deer in this clade were harvested proximate to herds A, B, C, F, and J (within 5 miles), but the phylogeny of the isolates suggests that the *M*. *bovis* infecting these white-tailed deer had strongly diverged from the isolates in these spatially proximate herds. Overall, there was no strong pattern of *M*. *bovis* genetic divergence with geographic distance among any subset of hosts, suggesting that the mechanism(s) of transmission was capable of dispersing *M*. *bovis* at a scale similar to the extent of the outbreak.

## Supporting Information

S1 Fig Bayesian Skyline Plot of Mean +/- 95% Highest Posterior Density (HPD) Interval of Effective Population Size of *Mycobacterium bovis* Isolates from Minnesota 2004–2009. Dark vertical dashed line is the median estimate of time of most recent common ancestor (MRCA) and light vertical dashed line is the lower 95% HPD estimate of time of MRCA. The plot begins at the 95% upper HPD estimate of time of MRCA.
(EPS)

S1 Table The Number of Herd Tests and Deer Tested and Number of bTB Infected Herds and Deer Identified Each Year. (DOCX)

S2 Table A. SNP Chart of Whole Genome Sequences of Isolates from Infected Animals in the Outbreak. B. SNP Statistics of Whole Genome Sequences of Isolates from Infected Animals in the Outbreak.
(XLSX)

S3 Table Model Selection by AICM of Molecular Clock and Demography Models of *M*. *bovis* Sequences from Minnesota and a Texas Beef Herd. (DOCX)

S4 Table Model Selection by AICM of Molecular Clock and Demography Models of *M*. *bovis* Sequences from the Minnesota Outbreak. (DOCX)

S1 Text Chronological Discovery of Infected Herds and Deer. (DOCX)

S2 Text Phylogenetic Analyses. (DOCX)
