# Supplementary material for: Descriptive Epidemiology and Whole Genome Sequencing Analysis for an Outbreak of Bovine Tuberculosis in Beef Cattle and White-Tailed Deer in Northwestern Minnesota
Source: PLoS One. 2016 Jan 19;11(1):e0145735. doi: 10.1371/journal.pone.0145735 (PMC4718535; doi:10.1371/journal.pone.0145735)
Supplement: S1 Table — (DOCX) [file pone.0145735.s002.docx]

**S1 Table. The Number of Herd Tests and Deer tested and Number of bTB Infected Herds and Deer Identified each Year.**

| **Month** | **2005** | 2006 | 2007 | 2008 | 2009 | 2010 | 2011 | 2012 |
| --- | --- | --- | --- | --- | --- | --- | --- | --- |
| **Number tested** | Herds = 50^a^  Deer = 474^b^ | Herds=329 SS Herds = 604^c^  Deer = 1,032 | Herds = 147 SS Herds = 947  Deer = 1,654 SS Deer =4000^c^ | Herds=155  Deer = 2,308 | Herds =17  Deer = 2,226 | Herds = 5  Deer = 2,089 | Herds = 2  Deer = 561 | Herds = 0  Deer = 323 |
| **bTB Positive Herds and Deer** | | | | | | | | |
| January |  | Deer (n=1) |  | Herd I |  |  |  |  |
| February |  |  | Deer (n=2) | Herds J, K | Deer (n=2) |  |  |  |
| March |  |  | Deer (n=2) | Deer (n=3) |  |  |  |  |
| April |  |  | Deer (n=2) | Deer (n=3) |  |  |  |  |
| May |  |  |  |  |  |  |  |  |
| June |  |  |  |  |  |  |  |  |
| July | Herd A^d^ |  |  |  |  |  |  |  |
| August |  |  |  |  |  |  |  |  |
| September | Herds B, C |  |  |  |  |  |  |  |
| October | Herd D | Herds F, G | Herd H | Herd L |  |  |  |  |
| November | Deer (n=1)^e^ | Deer (n=5) | Deer (n=5) |  | Deer (n=1) |  |  |  |
| December | Herd E |  |  |  |  |  |  |  |

^a^Number of whole herd tests (all animals >12 months and older in the herd) of cattle conducted that year for the investigation

^b^Number of deer tested in the area of the infected cattle herds

^c^Number of herds or deer tested for statewide surveillance (SS).

^d^Placement of herd in this chart represents the month the herd was declared Affected.

^e^Placement of deer in this table represents the month the deer was harvested.
