# Supplementary material for: Descriptive Epidemiology and Whole Genome Sequencing Analysis for an Outbreak of Bovine Tuberculosis in Beef Cattle and White-Tailed Deer in Northwestern Minnesota
Source: PLoS One. 2016 Jan 19;11(1):e0145735. doi: 10.1371/journal.pone.0145735 (PMC4718535; doi:10.1371/journal.pone.0145735)
Supplement: S3 Table — (DOCX) [file pone.0145735.s004.docx]

| \| **S3 Table. Model Selection by AICM of Molecular Clock and Demography Models of *M. bovis* Sequences from Minnesota and a Texas Beef Herd.** \| \| \| \| --- \| --- \| --- \| \| Model \| AICM \| se^d^ \|  \| Strict Clock Rate - Varying Effective Population^a^ - MN monophyletic^b^ \| 1018.107 \| 0.38 \| \| --- \| --- \| --- \| \| Strict Clock Rate - Constant Effective Population - MN monophyletic \| 1027.674 \| 0.40 \| \| Strict Clock Rate - Varying Effective Population \| 1042.085 \| 0.18 \| \| Relaxed Clock Rate^c^ - Varying Effective Population \| 1042.209 \| 0.30 \| \| Relaxed Clock Rate - Constant Effective Population \| 1049.975 \| 0.25 \| \| Relaxed Clock Rate - Varying Effective Population - MN monophyletic \| 1050.594 \| 0.33 \| \| Relaxed Clock Rate - Varying Effective Population - MN monophyletic \| 1057.466 \| 0.18 \| \| Strict Clock Rate - Constant Effective Population \| 1065.065 \| 0.39 \|  \| ^a^ Effective population size estimated with Bayesian skyline methods with 5 *a priori* groups [15]. \| \| --- \| \| ^b^ Models with prior that fixed all isolates from Minnesota as a monophyletic clade \| \| ^c^ Relaxed clock rate modeled with uncorrelated lognormal distribution [16]. \| \| ^d^ Standard errors estimated from 100 bootstrap replicates in Tracer v1.6 [18]. \| |
| --- | --- | --- | --- | --- | --- | --- | --- | --- | --- | --- | --- | --- | --- | --- | --- | --- | --- | --- | --- | --- | --- | --- | --- | --- | --- | --- | --- | --- | --- | --- | --- | --- | --- | --- |
