# Supplementary material for: Descriptive Epidemiology and Whole Genome Sequencing Analysis for an Outbreak of Bovine Tuberculosis in Beef Cattle and White-Tailed Deer in Northwestern Minnesota
Source: PLoS One. 2016 Jan 19;11(1):e0145735. doi: 10.1371/journal.pone.0145735 (PMC4718535; doi:10.1371/journal.pone.0145735)
Supplement: S4 Table — (DOCX) [file pone.0145735.s005.docx]

| **S4 Table. Model selection by AICM of Molecular Clock and Demography Models of *M. bovis* Sequences from the Minnesota Outbreak.** | | |
| --- | --- | --- |
| Model | AICM | se^c^ |
| Strict Clock Rate - Varying Effective Population^a^ | 827.73 | 0.035 |
| Strict Clock Rate - Constant Effective Population | 843.84 | 0.050 |
| Relaxed Clock Rate^b^ - Varying Effective Population | 844.51 | 0.089 |
| Relaxed Clock Rate - Constant Effective Population | 868.16 | 0.155 |
| ^a^ Effective population size estimated with Bayesian skyline methods with 5 *a priori* groups [15]. | | |
| ^b^ Relaxed clock rate modeled with uncorrelated lognormal distribution [16]. | | |
| ^c^ Standard errors estimated from 100 bootstrap replicates in Tracer v1.6 [18]. | | |
