# Supplementary material for: Descriptive Epidemiology and Whole Genome Sequencing Analysis for an Outbreak of Bovine Tuberculosis in Beef Cattle and White-Tailed Deer in Northwestern Minnesota
Source: PLoS One. 2016 Jan 19;11(1):e0145735. doi: 10.1371/journal.pone.0145735 (PMC4718535; doi:10.1371/journal.pone.0145735)
Supplement: S1 Text — (DOCX) [file pone.0145735.s006.docx]

**S1 Text. Chronological Discovery of Infected Herds and Deer.**

**2005**

Index Herd (Herd A) - This beef herd was tested in May 2005; 63 animals had a positive test response on the CFT test (10.8%). The CCT test was used as the follow up test on these animals and 21 animals were classified as Suspect or Reactor animals, euthanized and submitted to the Minnesota Veterinary Diagnostic Laboratory for necropsy and diagnostic testing in June 2005.

Six bTB infected female cows were identified; three, five (two cows), six, eight, and 10 years old. The herd was declared ‘Affected’ in July 2005. Four additional animals were condemned at slaughter with gross lesions of bTB but no diagnostic follow up was done (the animals ranged in age from three to seven years). An epidemiological investigation was initiated and two of the four fence line contact herds had bTB positive animals. A year later, the two remaining contact herds were tested again and one of these herds was discovered to have TB positive animals. Cattle movements in and out of the herd were traced back seven years including private sales and animals sold at a local sales barn.

Private sales from the index herd included 13 Minnesota producers and 9 producers in four other states that had purchased cattle; 6 Minnesota producers and 19 producers from six other states sold animals to this herd. Animals purchased from the index herd in 2003 by a local herd owner were found infected with bTB. No infected animals were identified in herds that had sold animals to the index herd owner.

Herd B was a commercial beef herd tested for bTB as animals in that herd were pastured adjacent to the Herd A. Herd B raised heifers for replacements and bought bulls from local farmers in northwestern Minnesota. Three animals were identified as infected in the herd; two 14 year old cows born on the farm and one 5 year old bull purchased in 2002.

Herd C was a commercial beef herd tested for bTB as animals were pastured adjacent to Herd A. Herd C raised all its own replacement heifers and bought bulls locally. One infected animal was identified in this herd; a 1 ½ year old heifer born on the farm.

Herd D was identified in the investigation for bTB testing as the producer had purchased animals from the Herd A in February 2003. Herd D was started in 2002 and included animals from several states acquired from the same network of breeders as Herd A. Fifty-three animals purchased by this farmer from the Herd A owner (exposed animals) were indemnified and slaughtered. On enhanced carcass inspection at slaughter, samples were collected for bTB testing from seven animals; three 3 year olds were found infected with bTB. The remainder of the herd was declared ‘Affected’).

Herd E was identified for bTB testing as it sold a bull to Herd C. Herd E also purchased heifers through the sales barn from Herd C in Feb. 2001. Bulls were purchased from northern Minnesota. A smaller component of the herd was purebred and animals were purchased from several states. One 10 year old beef cow was found infected as a result of the herd bTB test. This animal was purchased as a three year old in a group of 50 animals from a private herd buyout after the death of the owner. Herd E was declared ‘Affected’. Enhanced slaughter surveillance with sample collection and testing found two 10 month old purebred Angus heifers born on the farm infected with TB.

First Infected Deer - In 2005, the Minnesota Department of Natural Resources collected samples from 474 hunter-harvested deer within 15 miles of the first five infected cattle herds. This surveillance effort discovered one positive deer within a mile of Herd A.

**2006**

Targeted deer surveillance - Following the discovery of a bTB infected deer in 2005, landowner shooting permits were provided to cattle producers with infected cattle herds and producers adjacent to infected farms in January and February 2006; 90 deer were sampled and one additional infected deer was detected. Both bTB positive deer had the same strain of bTB as the cattle.

Herd F was tested for bTB in 2005 as a herd adjacent to the Herd A and was negative. This was a small commercial beef herd that purchased bulls locally, raised their own heifers for replacements and also bought dairy steers from a neighbor for feeding. A second herd test of Herd F in the fall of 2006 found the herd had one infected animal, a two year old cow born on the farm that tested negative for bTB in 2005.

Herd G was within ten miles of Herd A and was TB tested in the fall of 2006. This was a small commercial beef herd that replaced cows with heifers raised on the farm and purchased bulls locally. One positive animal was found infected in the herd; a 2 year old cow born on the farm.

Hunter-harvested deer surveillance - In the fall of 2006 five infected animals were detected out of 942 deer tested, bringing the total number of deer found infected at that time to seven.

**2007**

Targeted deer surveillance - Efforts intensified in 2007 with the addition of agency sponsored sharpshooting around the known bTB infected cattle herds between February and late March; 488 deer were sampled and six found bTB infected.

Herd H was a commercial beef herd found infected with the first part of a herd test in 2007. This herd tested negative for bTB in 2006 with a trace investigation, the owner had purchased animals from Herd E. The second herd test for Herd H was scheduled in 2007 as the herd was also within 10 miles of Herd E. A group of heifers was tested first and one 18 month old animal was found infected. The rest of the herd was not tested. In early 2008, a steer from this herd in the same age class as the infected heifer was found infected at slaughter. The animal had been sold by the herd owner in 2007 and fed out in a Nebraska feedlot.

Hunter-harvested deer surveillance - In the fall of 2007, 1,166 hunter-harvested deer were sampled for bTB; five animals were found infected.

**2008**

Herd I was tested for bTB the previous two years as summer pasture land used by this herd for grazing was within ten miles of a previously infected herd. Herd I was a commercial beef herd that regularly added animals to the herd from the local sale barn. The TB test identified one infected five year old cow that had been purchased from the sales barn in either 2003 or 2004.

Herd J was a small commercial beef herd that had also been tested for bTB in each of the past two years as it was adjacent to Herd B. Herd J raises its own replacement heifers and buys bulls locally. The third TB test identified a 10 year old and a 12 year old infected cow. Investigations of animal movement in Herds I and J found that heifers purchased at the local sales barn in February 2004 by Herd I were from Herd J. It is unknown if either of these heifers could be the animal found infected in Herd I. No animal identification was recorded in the sale or found on the animals to definitively identify them as from Herd J.

Herd K identified an 8 year old bTB infected cow on its second herd test. This herd was initially identified for testing as it was within 10 miles of the first group of TB infected herds. This is a small commercial beef herd which keeps replacements from the herd and buys bulls locally.

Targeted deer surveillance – In March and April 2008, 937 deer were collected and tested for bTB through agency sponsored culling; six positive deer were identified. An additional 125 deer were harvested by landowner shooting permits in the bTB affected area; no positive deer were identified.

Herd L was found infected in October 2008 when animals were taken to slaughter as part of a voluntary buyout program to reduce the livestock population in the TB affected area of northwestern Minnesota. The buyout agreement required the herd to be slaughtered. Herd L had initially been identified for testing as an area herd and tested negative for bTB in 2006 and 2007. This beef herd had both purebred and commercial animals and did bring animals into the herd from other states. Three adult animals ages 13, 7, and 5 were identified in 2008 and an additional nine yearlings were found infected at slaughter in January 2009.

Hunter-harvested deer – In the fall of 2008, 1,246 deer were collected and tested; no deer were found positive for bTB

**2009**

Targeted deer surveillance – In February-April, 738 deer were collected and tested through agency sponsored culling, 2 deer were positive for bTB.

Hunter-harvested deer – In the fall of 2009, 1,488 deer collected and tested; 1 deer positive for bTB.

**2010-2012**

In each of these years, hunter harvested deer were sampled and tested with no animals found positive for bTB. In addition, agency sponsored culling in 2010 also yielded no additional positive cases.
