# Supplementary material for: Descriptive Epidemiology and Whole Genome Sequencing Analysis for an Outbreak of Bovine Tuberculosis in Beef Cattle and White-Tailed Deer in Northwestern Minnesota
Source: PLoS One. 2016 Jan 19;11(1):e0145735. doi: 10.1371/journal.pone.0145735 (PMC4718535; doi:10.1371/journal.pone.0145735)
Supplement: S2 Text — (DOCX) [file pone.0145735.s007.docx]

**S2 Text. Phylogenetic analyses**

**Model specification and comparison**

Model comparison was conducted using Akaike’s information criterion through Markov chain Monte Carlo (AICM; [17] on MCMC chains of 100 million generations and a 10% burn-in for all combinations of molecular clock model (strict and relaxed) and demographic model (coalescent constant population size and Bayesian skyline with 5 *a priori* coalescent intervals. The site substitution model used for comparisons was GTR+Γ with the C-T rate fixed and relative rates estimated for all other nucleotide substitutions. AICM values were calculated using Tracer v1.6 and 100 bootstrap replicates were used to generate standard errors (se) [18].

Based on observed SNP change rates of a *M. bovis* isolated from outbreaks throughout the United States (unpublished data), an informative lognormal prior was used for the molecular clock rate (untransformed mean = 0.005, untransformed sd = 0.3) . All other priors were the default priors in BEAST (https://code.google.com/p/beast-mcmc/wiki/ParameterPriors).

Model comparison for phylogenetic models of the Minnesota and Texas isolates also included models with an additional prior that fixed the Minnesota isolates as monophyletic. The best fit model to the Minnesota and Texas isolate sequences was a strict molecular clock with a Bayesian skyline demographic model and the Minnesota isolates as a monophyletic clade (S3 Table).

Model comparison using only the Minnesota isolates also found that a strict clock rate with Bayesian skyline demographic model for effective population size was the best fit to the data (dAICM >10) (S4 Table). The demographic reconstruction from the best fit model (5x 100 million iteration MCMC chains with 10% burn-in) demonstrated an expanding effective population size through the Minnesota outbreak (S1 Figure).
